# In vitro machine learning-based CAR T immunological synapse quality measurements correlate with patient clinical outcomes

**DOI:** 10.1371/journal.pcbi.1009883

**Published:** 2022-03-18

**Authors:** Alireza Naghizadeh, Wei-chung Tsao, Jong Hyun Cho, Hongye Xu, Mohab Mohamed, Dali Li, Wei Xiong, Dimitri Metaxas, Carlos A. Ramos, Dongfang Liu

**Affiliations:** 1 Department of Pathology, Immunology and Laboratory Medicine, Rutgers University-New Jersey Medical School, Newark, New Jersey, United States of America; 2 Center for Immunity and Inflammation, New Jersey Medical School, Rutgers-The State University of New Jersey, Newark, New Jersey, United States of America; 3 Center for Inflammation and Epigenetics, Houston Methodist Research Institute, Houston, Texas, United States of America; 4 Department of Computer Science, Rutgers University, Piscataway Township, New Jersey, United States of America; 5 Department of Medicine, Baylor College of Medicine, Houston, Texas, United States of America; University of Pittsburgh, UNITED STATES

## Abstract

The human immune system consists of a highly intelligent network of billions of independent, self-organized cells that interact with each other. Machine learning (ML) is an artificial intelligence (AI) tool that automatically processes huge amounts of image data. Immunotherapies have revolutionized the treatment of blood cancer. Specifically, one such therapy involves engineering immune cells to express chimeric antigen receptors (CAR), which combine tumor antigen specificity with immune cell activation in a single receptor. To improve their efficacy and expand their applicability to solid tumors, scientists optimize different CARs with different modifications. However, predicting and ranking the efficacy of different "off-the-shelf" immune products (e.g., CAR or Bispecific T-cell Engager [BiTE]) and selection of clinical responders are challenging in clinical practice. Meanwhile, identifying the optimal CAR construct for a researcher to further develop a potential clinical application is limited by the current, time-consuming, costly, and labor-intensive conventional tools used to evaluate efficacy. Particularly, more than 30 years of immunological synapse (IS) research data demonstrate that T cell efficacy is not only controlled by the specificity and avidity of the tumor antigen and T cell interaction, but also it depends on a collective process, involving multiple adhesion and regulatory molecules, as well as tumor microenvironment, spatially and temporally organized at the IS formed by cytotoxic T lymphocytes (CTL) and natural killer (NK) cells. The optimal function of cytotoxic lymphocytes (including CTL and NK) depends on IS quality. Recognizing the inadequacy of conventional tools and the importance of IS in immune cell functions, we investigate a new strategy for assessing CAR-T efficacy by quantifying CAR IS quality using the glass-support planar lipid bilayer system combined with ML-based data analysis. Previous studies in our group show that CAR-T IS quality correlates with antitumor activities *in vitro* and *in vivo*. However, current manually quantified IS quality data analysis is time-consuming and labor-intensive with low accuracy, reproducibility, and repeatability. In this study, we develop a novel ML-based method to quantify thousands of CAR cell IS images with enhanced accuracy and speed. Specifically, we used artificial neural networks (ANN) to incorporate object detection into segmentation. The proposed ANN model extracts the most useful information to differentiate different IS datasets. The network output is flexible and produces bounding boxes, instance segmentation, contour outlines (borders), intensities of the borders, and segmentations without borders. Based on requirements, one or a combination of this information is used in statistical analysis. The ML-based automated algorithm quantified CAR-T IS data correlates with the clinical responder and non-responder treated with Kappa-CAR-T cells directly from patients. The results suggest that CAR cell IS quality can be used as a potential composite biomarker and correlates with antitumor activities in patients, which is sufficiently discriminative to further test the CAR IS quality as a clinical biomarker to predict response to CAR immunotherapy in cancer. For translational research, the method developed here can also provide guidelines for designing and optimizing numerous CAR constructs for potential clinical development.

**Trial Registration:** ClinicalTrials.gov NCT00881920.

## Author summary

Adoptive transfer of chimeric antigen receptor (CAR)-modified immune cells (including CAR-T and CAR-NK cells) have revolutionized the treatment of cancer with success in clinical trials treating multiple myeloma, leukemia, sarcoma, and neuroblastoma. However, CAR-modified immune cells (particularly CAR-T cells) must form a functional immunological synapse (IS) with their susceptible tumor cells to be effective in clinics. Currently, there are no effective biomarkers to predict CAR efficacy *in vivo*. In this study, we develop a state-of-the-art machine learning (ML) detection, and segmentation method to measure the quality of the CAR-T cell IS using CAR-T samples from patients. We automate the IS quality analysis to develop effective prognostic applications of CAR-T therapies for cancer patients. The fast, easy-to-implement Synapse Predicts Efficacy (SPE) assay we propose will streamline CAR development and selection, ultimately optimizing clinical outcome(s) for patients undergoing these rapidly evolving immunotherapies. This technology can lead to development of fast and easy tools to predict CAR-T cell effectiveness in cancer patients.

## Introduction

Adoptive T cell-based immunotherapy with chimeric antigen receptors (CAR) has shown to be effective for treating refractory blood cancers [1]. However, predicting the effectiveness of CAR-T cells represents an unsolved problem in the field of immunotherapy [2–4].

Different CARs are being actively generated with different modifications from different research laboratories [5–7]. It is not practicable to test all the different modifications of CARs in pre-clinical assays or clinical trials due to the high costs, time constraints, and complexity of CAR manufacturing. Thus, it is essential that these different CARs can be evaluated for their efficacy and safety in a cost-effective, timely, and reproducible manner *in vitro*.

Previous studies show that the quality of CAR immunological synapse (IS) can predict the effectiveness of CAR cells [4,8,9]. The method for imaging the IS has been originally described on interactions between T-cell and antigen-presenting cells (APCs) [5,10–14]. Findings from this method, such as the structure, function, and signaling cascades at the synapses, are confirmed with a glass-supported planar lipid bilayer [8]. The glass-supported planar lipid bilayer system can emulate the target cell activities. The activities of CAR cells stimulated with different ligands on the glass-supported planar lipid bilayers can aid the evaluation of synapses with high-resolution images [14,15–17], which can lead to measuring the effectiveness of CAR-T cells [4,8].

Currently available strategies to pre-evaluate the effectiveness of CAR-T cells include cytokine secretion (TNF-α and IFN-γ), standard *Cr*^51^ release assays, proliferation assays, CD4/CD8 ratios, *in vitro* long-term killing assays, severe combined immunodeficiency (SCID) mouse models, and *in vivo* imaging systems [4,18–25]. Conventional *in vitro* cytokine productions, CD4/CD8 ratios, and *Cr*^51^ release assays cannot accurately predict CAR-T efficacy *in vivo*. The standard methods for predicting CAR cell performance in patients include the long-term killing assays and *in vivo* animal models [4]. However, the long-term killing assays and *in vivo* mouse models are time-consuming and costly. The quality of the CAR cell IS, in stark contrast, correlates with superior long-term killing efficiency and proliferation ability, as determined by both *in vitro* long-term killing assays and *in vivo* mouse models and imaging systems [8]. One caveat of this SPE assay developed in the previous studies to quantify the IS quality depends on the manual quantification of parameters found within the IS (e.g., F-actin accumulation, lytic granule polarization), which is time-consuming, labor-intensive, and inconsistent among different experimenters with limited IS numbers for quantification (usually less than 100 IS numbers). Additionally, the IS quality measurements have not been evaluated using CAR-T cells directly generated from actual patients.

In this study, we developed an automatic, machine learning (ML)-based approach to quantify CARs within the IS by instance segmentation through high-resolution image of interaction between CAR-T and its cognate tumor antigen only reconstituted on the glass-supported planar lipid bilayer. We have chosen to compare CAR-T cells from two different patients (i.e., responder *vs*. non-responder) with the identical CAR construct throughout the study as proof of concept that differences in CAR IS quality translate into measurable differences in clinical outcomes. The particular problems we solved in this study include: 1) classifying the objects, 2) separating them from neighboring cells, and 3) quantifying more than thousands of IS numbers per sample from patients automatically. The main difficulties for separating the neighboring cells from each other include low contrast of cell boundaries, background noise (impurities), adhesion, and cell clustering. The most effective and accurate method we developed here is incorporating object detection into segmentation. This method plays an important role in biomedical data analysis, such as cell migration study [26] and cell nuclei detection [27]. Detection and segmentation of the cells in microscopic images can be more effectively performed in multi-scale cell instance segmentation [28,29]. An important feature that helps these methods is their ability to distinguish objects based on their global features and not local pixel-level information.

In summary, this study provides an efficient, cost-effective, easy-to-use, automated approach to quantify the quality of CARs within the IS, which can be used to support and optimize the clinical use of CARs in the field of immunotherapy.

## Results

### Characterizations of CAR-T cells from two types of patients (a responder and a non-responder of Kappa-CAR-T treatment)

**Fig 1:**
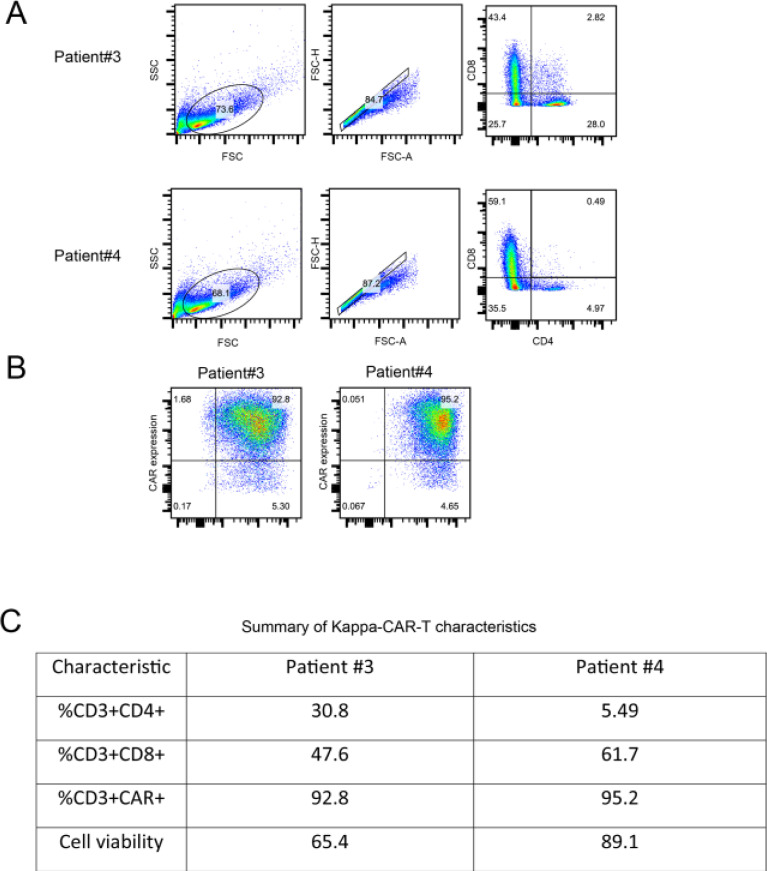
Comparable CAR expressions between patient #3 and patient #4. PBMCs from patients #3 and #4 were transduced with the kappa-CAR retrovirus, respectively. The ratio and expression (MFI) of CD8 and CD4 subsets are calculated. (A) Flow cytometry analysis of CD8 and CD4 positive population from patients #3 and #4. (B) The ratio of CD3 and CAR positive subsets is calculated. (C) Different subsets of CD3^+^ T cells and viability are summarized. Data are pooled from at least two independent experiments.

Responders include patients with complete (CR) and partial remission (PR), whereas non-responders have stable (SD) or progressive disease (PD). A recent clinical trial (ClinicalTrials.gov NCT00924326) determined that the serum levels of 41 different proteins (except for IL-15 and IL-10) were not significantly different in responders *vs*. non-responders [30]. This is consistent with our recent clinical trial data on kappa-CAR-T cells [31]. We first characterized the CAR-T cells from patient #3 and patient #4. The clinical characterizations of patients #3 and #4 are described in the Materials and Methods. We compared the subsets of CD4 positive and CD8 positive lymphocytes between patient #3 and patient #4. The percentages of CD4 and CD8 subsets are comparable between patient #3 and patient #4 (Fig 1A). Similar results were obtained in the percentage of CAR^+^ and CD3^+^ T cells (Fig 1B). However, a higher percentage of CD8^+^ T cells and viability from patient #4 is observed compared to patient #3 (Fig 1C). In summary, the percentage of CAR positive T cells, CAR molecular expression on individual CAR-T cells, and viability are slightly higher from patient #4 than from patient #3.

### CAR IS formation on the glass-supported planar lipid bilayer

**Fig 2:**
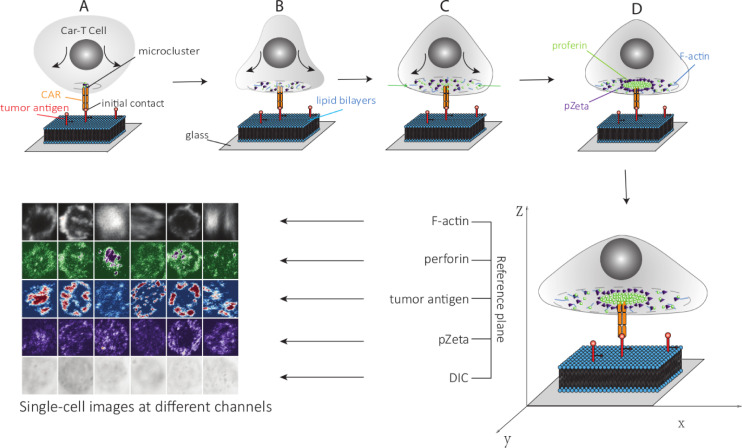
The model shows the process of perforin and pZeta cluster formation, and accumulation of F-actin formation after the initial contact of the CAR-T and planar lipid bilayer. (A) At the initial contact of the CAR with the tumor antigen, micro clusters are formed around the receptor, and the cell starts to spread. (B) The cell spread, and multiple microclusters form. (C) After the cell spread, F-actin polymerizes at the cell periphery. The perforin and pZeta are transported toward the cell center along with F-actin. (D) Perforin and pZeta populate the actin-sparse center and form a cluster. In the experiment, we labeled the different substances with different colors, and different channels of images were obtained using different lasers. We use six single-cell samples in five channels using the best Z position.

We used fluorescently labeled Kappa protein to stimulate CAR-T cells on lipid bilayers. As described in [32], fluorescently conjugated antibodies-stained cells against perforin (deltaG9, Thermo Scientific) and pZeta (phosphor-Y83, Abcam). F-actin was stained by Alexa Fluor 532-conjugated phalloidin (Life Technologies). After preparing the glass-supported planar lipid bilayer system, we take high-resolution 3D images to study the IS for CAR-T cells (Fig 2).

From the 3D confocal images, we have seen some important properties of the CAR IS. These properties include Kappa protein on the focal plane of the glass-supported planar lipid bilayer, which can mirror the CAR-modified cells’ distribution of CAR proteins. The effectiveness of the prediction of CAR-modified cells is measured by multiple parameters, including the amount of Kappa (reflecting the amount of tumor antigen accumulation with CAR in the IS), accumulation of F-actin within CAR in the IS, the polarization of lytic granules (LGs) within CAR in the IS, and distribution of key signaling molecules (pZeta) within the IS. The measurement is confirmed from both tumor cell numbers and CAR-modified cell proliferation during a long-term killing assay and in vivo efficacy in a mouse xenograft model [8].

### The overall model of instance segmentation for kappa-CAR-T cells

The multi-scale cell instance segmentation is developed [28,29] to address the challenges of the data: 1) The cells are stacked together or clustered together, and the cell boundary is hard to differentiate, 2) The cells are in irregular shapes, and 3) cell occlusion. The masked objects can easily be used to generate boundaries (contours) around their respective cells. The contours are required to determine the area of each cell, which is used for statistical analysis. In the end, we combine all channel information to produce more knowledge from the model.

**Fig 3:**
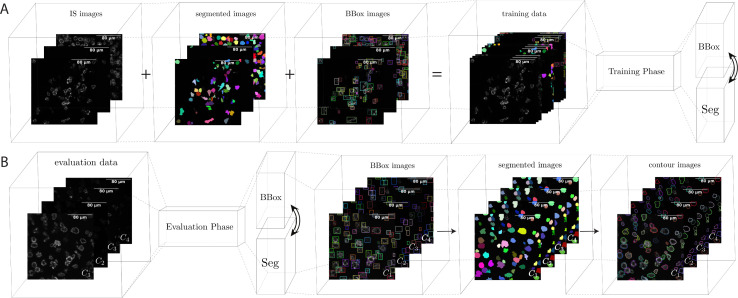
The overall model of instance segmentation for CAR-T cells using multi-scale cell instance segmentation. (A) Demonstrates the training phase. In this phase, CAR-T IS images are used for training sets. (B) Shows the model in the evaluation phase. In this phase, each sample has five channels, of which four of them are applicable for evaluation. Channel 3 is used to select the best Z slide, and Channel 1 provides the best possible representation of the CAR-T IS. From Channel 1, the network produces bounding boxes, instance segmentation, and contours. The generated masks and contours are applied on all channels for statistical analysis.

Fig 3 shows the overall model we use for instance segmentation on our data. Similar to other supervised ML methods, we need two different phases. The first phase is called training, in which the available labeled data is used to train the ANNs. The second phase is the evaluation phase (also called the testing phase). In this step, we use the trained model to perform the real evaluation on CAR-T IS images. The network produces bounding boxes, instance segmentation, and contours in the evaluation phase. The generated masks and contours are applied on all channels for statistical analysis. Based on the requirements for statistical analysis, intensity parameters other than total intensity can be added such as intensities of the borders and intensities of the segmentations without borders.

Fig 3A demonstrates the training phase. To create a training set, the images of the IS from CAR-T cells were collected and segmented. The images are carefully segmented and annotated manually. Our software used the segmented images to automatically generate bounding boxes. The segmented images and bounding boxes were used to train the artificial neural networks (ANNs). Fig 3B shows the model in the evaluation phase. Among the five channels we received from imaging of glass-supported planar lipid bilayers, the first four channels are intrinsically applicable for evaluations (DIC images are not used in this process). Each channel contains a limited number of slides that show the image with different intensity modes. A pre-processing step was applied to the tumor antigen (channel 3) image signaling to obtain the focal plane of immunological synapse on the glass-supported planar lipid bilayer. Using this pre-processing step, we selected slide Z with the best intensity. Next, F-actin (Channel 1) was used to perform multi-scale cell instance segmentation, which extracts the segmented masks for each cell object.

**Fig 4:**
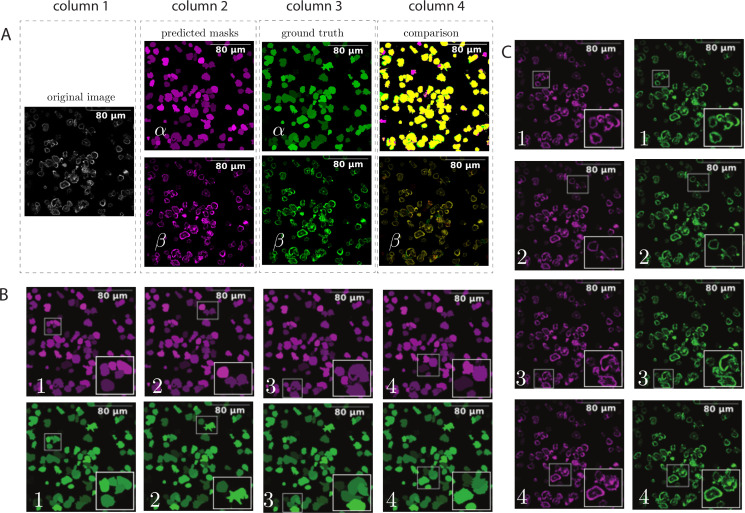
Comparison of generated instance segmentation masks in the evaluation phase with their ground truths. We applied colormaps ’Magenta’, ’Green’, and ’Yellow’ for better representation of the images. Different shades are used to separate the cells from each other. Four different zooming areas are selected for analysis. The images with the same number point to the same zooming area.

After successfully generating the bounding boxes, instance segmentation, and contours, we further compared the ground truths with the segmentation produced by ANNs. Fig 4 illustrates the comparison of generated instance segmentation masks in the evaluation phase with their respective ground truths. In Fig 4A, the test sample is shown in the first column, which is in its original grayscale format. The second column shows the prediction for instance segmentation of cells in the evaluation phase. The third column shows the manually masked cells with the help of a human expert. In the fourth column (comparisons), the generated masks are overlapped with ground truth to represent the method’s accuracy. The α images show all the available masks. The β images show the pixels that are overlayed with the masks. All the pixels that do not belong to segmented masks are removed. In Fig 4B, we zoom into different parts of Fig 4A to observe the model’s performance compared to a human expert. In Fig 4C, we zoom into the same parts of the images to observe the pixels. In Fig 4B and Fig 4C, the images that share the same number, point to the same zoomed areas.

We encountered three scenarios from overlapping the ground truth with the predicted masks. The ground truth masks and predicted segmentation masks were very close in the first scenario. Naturally, the evaluation scores for these areas are high. In the second scenario, small discrepancies were visible, and in the third scenario, the ground truth masks, and predicted masks were not as close. These types of discrepancies can potentially lower the evaluation scores. We can focus on three main aspects to prevent segmentation errors and improve the evaluation scores as follows: 1) when creating training data, we should prevent pixel errors as much as possible. If the training data has false negative or false positive masks, it can adversely affect the accuracy of the predicted masks. 2) We can increase the amount of training data by increasing labor resources. In general, more training data can lead to higher accuracy. 3) Improve the underlying infrastructure of bounding box detection and instance segmentation algorithms. This is possible by following the improvements of related algorithms in ML.

### Instance segmentation for CAR-T cell model training and testing

Previous studies have shown that ANNs are superior to traditional optimization methods as they automatically extract the correct features from provided data to perform tasks such as detection, estimation, and classification [28,33–35]. Multi-scale cell instance segmentation handles cells at different scales. Combining this approach with separating bounding box detection and instance segmentation creates one of the most effective ANNs for detecting CAR-T cells. In the following, we compare instant segmentation (InstSeg) to three more methods, DCAN [36], CosineEmbedding [26], and Mask R-CNN [37], to establish the effectiveness of the method.

**Fig 5:**
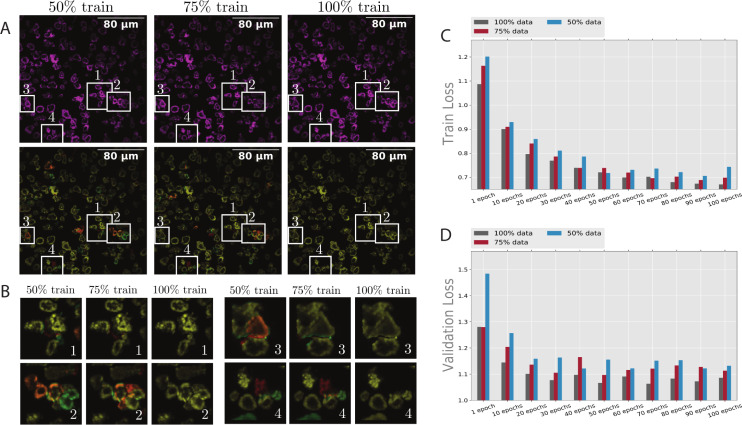
The comparison of the model’s loss with different sets of training data. (A) Represents a test image sample in the evaluation phase using the defined trained networks. In (B), four different zoomed areas are selected for analysis. Images with similar numbers point to the same boxes in (A). These images present the effect of having access to more training data and its role in removing discrepancies. (C) Shows the training loss, and (D) Shows the validation loss from 0 to 100 training iterations with 100% of the training data. As expected, the training loss shows a more predictable pattern than validation loss.

Because of the expertise required to separate CAR-T cells and the sensitivity to correctly perform this operation, labor scarcity is a major hurdle to creating training data. There are overall 156 manually masked images. For the experiments, we use 60% of the 156 images for training (93-image dataset), 20% for testing (31-image dataset), and 20% for validation (32-image dataset), as shown in Fig 5. To evaluate the effectiveness of the proposed method for number of the training sets, we experiment on 1–50% of the training set (≈ 47 images), 2–75% of the training set (≈ 70 images), 3–100% of the training set (≈ 93 images).

We optimized the model parameters using Adam optimizer [38] with 0.0001 as the initial learning rate in the training process. To help with attaining better generalization, augmentation methods such as random expansion, cropping, flipping, contrast distortion, and brightness distortion are deployed. We stop the training after 100 epochs. The model predicts the segmentation masks of each cell. The masks will be transformed into contours to collect statistics. The effects of training with different datasets and different epochs (iterations) are shown in Fig 5. Fig 5A represents a test image sample in the evaluation phase to show the trained networks with different training sets. The first row shows the pixels under segmented areas. This means that all the pixels that do not belong to a segmented mask are removed. We performed this operation on the image to make comparisons easier. The generated masks are overlapped with ground truth pixels in the second row. We use colormap ’Magenta’ for the predicted segmentation and colormap ’green’ for manually masked images. Therefore, the closer the color is yellow (combination of ’Magenta’ and ’green’), the stronger the accuracy. On the other hand, if the pixels are closer to ’green’ and ’Magenta’, they show discrepancies between predictions and their respective ground truths.

In Fig 5A, we observe that with more training data, the accuracy increases (pixels get closer to yellow), but the model still performs quite well with a lower number of training sets. In Fig 5B, four different zoomed areas are selected to analyze discrepancies better. These images present the effect of having access to more training data and its role in removing discrepancies. In Fig 5B, images with similar numbers point to the same boxes in Fig 5A. To examine the effect of epochs on training, we present two figures. The training loss is shown in Fig 5C, and the validation loss is shown in Fig 5D. The plots show that the training converges to a certain point as it gets closer to epoch 100. Overall, Fig 5 demonstrates that while full training has the least validation and training loss, the model is resilient for smaller datasets and shows relatively close results.

The upper part of the Table 1 shows evaluation results for BBox evaluation. When the method is compared to DCAN with 75% of the data, instance segmentation is 30.78% better for AP@0.7. Respectively with 100% of the data, instance segmentation is at least 18.53% better for AP@0.5. Because of the low amount of training data, CosineEmbedding is not competitive and, on average, showed 60.7% worse results across all scenarios. Compared to Mask R-CNN, instance segmentation is 17.71% better for AP@0.7 with 75% of the data. Respectively, instance segmentation is at least 2.1% better for AP@0.5 with 100% of the data.

**Table 1:** The evaluation accuracy (%) for bounding box generation and instance segmentation. The upper part of the table is related to object detection (bounding boxes), and the lower part is related to instance segmentation. We used DCAN, CosineEmbedding, and Mask R-CNN for the other ANN architectures. When the entry is not applicable, dash (-) is used.

BBox Evaluation	50%-data AP@0.5	50%-data AP@0.7	50%-data IOU@0.5	50%-data IOU@0.7	75%-data AP@0.5	75%-data AP@0.7	75%-data IOU@0.5	75%-data IOU@0.7	100%-data AP@0.5	100%-data AP@0.7	100%-data IOU @0.5	100%-data IOU @0.7
DCAN	53.20	29.48	-	-	54.01	30.87	-	-	55.97	32.76	-	-
CosineEmbedding	8.70	0.82	-	-	11.24	1.14	-	-	13.26	2.40	-	-
Mask R-CNN	70.54	42.97	-	-	71.03	43.94	-	-	72.40	45.37	-	-
InstSeg	**74.19**	**55.06**	-	-	**74.22**	**61.65**	-	-	**74.50**	**62.14**	-	-
Segmentation Evaluation	50%-data AP@0.5	50%-data AP@0.7	50%-data IOU@0.5	50%-data IOU@0.7	75%-data AP@0.5	75%-data AP@0.7	75%-data IOU@0.5	75%-data IOU@0.7	100%-data AP@0.5	100%-data AP@0.7	100%-data IOU @0.5	100%-data IOU @0.7
DCAN	58.1	23.84	70.37	78.72	64.73	24.71	73.59	82.64	65.50	28.57	72.38	80.38
CosineEmbedding	21.19	0.64	59.60	74.70	23.08	8.18	64.51	74.72	23.97	5.27	63.21	74.15
Mask R-CNN	72.26	51.77	77.95	82.69	72.92	57.69	78.43	83.61	73.51	57.73	78.07	83.56
InstSeg	**74.90**	**56.21**	**80.37**	**84.60**	**74.94**	**63.03**	**81.06**	**84.89**	**75.14**	**63.43**	**81.09**	**84.93**

The lower part of the Table 1 shows evaluation results for segmentation evaluation. Similar to the BBox evaluation, the InstSeg is on average 54.22% better than CosineEmbedding across all scenarios. Compared to the other methods, when the method is compared to DCAN, InstSeg is at least 2.25% better for IOU@0.7 with 75% of the data and at most 38.32% better for AP@0.7 with 75% of the data. On average, InstSeg performed 15.08% better across all evaluation scenarios. Compared to Mask R-CNN, instance segmentation is utmost 5.34% better for AP@0.7 with 75% of the data. Respectively, instance segmentation is at least 1.28% better for IOU@0.7 with 75% of the data. On average, InstSeg had 2.86% better accuracy compared to Mask R-CNN.

### Differentiating between CAR-T cells from actual patients using instance segmentation

The purpose of the proposed model is to extract the most useful information from the data to differentiate between different sets. Using the trained model, we detected single cells in the cell images of patient #3 and patient #4 (see Table 2) and generated their masks. Since we are dealing with CAR-T IS, we evaluate the segmented areas. We detected a total of 2127 cells belonging to patient #3 and 2404 cells belonging to patient #4. For analysis, we use the total intensity of the cells of the two patients, as described in Section 3.4.

**Table 2:** Characteristics of patients with NHL or CLL [31].

Patient Characteristics	Age	Sex	Diagnosis	Previous therapies	Cytokines in CART culture	Time from last chemo. treatment	Pre-CART *CTX^A^*	DL	*CAR*^+^cells in product (%)	*CAR*^+^T cells/*m*^2^ admin.	No. of infusions	Best response
P3	55	M	FL/DLBCL	R-CHOP, R-ICE, R-BEAM/ASCT rituximab	IL-7/IL-15	16 wk	No	3	85	1.7×10^8^	6	*CR*×6 *wk*
P4	69	M	DLBCL	R-CHOP, R-BEA M/ASCT, R-bendamusti, ne/lenalidomide, R-ibrutinib, R-ESHAP,	IL-7/IL-15	14 d	No	3	93	1.9×10^8^	1	NR

**A** Low-dose CTX (12.5 mg/kg). admin., administered; chemo., chemotherapy; DL, dose level; MCL, mantle cell lymphoma; R-, Rituximab; CHOP, cyclophosphamide, doxorubicin, vincristine, prednisone; 2CDA, cladribine; BEAM, carmustine, etoposide, cytarabine, melphalan; FCR, fludarabine, cyclophosphamide, Rituximab; ICE, ifosfamide, carboplatin, etoposide; TTR, paclitaxel, topotecan, Rituximab; hCVAD, hyperfractionated cyclophosphamide, vincristine, cytarabine, doxorubicin, dexamethasone; ESHAP, etoposide, methylprednisolone, cytarabine, cisplatin; NR, no response.

Next, we compared IS quality between CAR-T therapy responders and non-responders, using relapsed or refractory + non-Hodgkin lymphoma/chronic lymphocytic leukemia (B-CLL) as test cases. To compare the IS quality between different types of patients (responders and non-responders), we provided the cumulative probability distribution and histogram plots for the same information extracted from IS from CAR-T cells. The histogram plots the frequency distribution (y-axis) as the binned data set (x-axis) function. The cumulative probability distribution displays the distribution of the data set from the smallest (from the left on the x-axis) to the greatest value (at the right of the x-axis) and provides the probability (y-axis) of whether a particular value will occur at or less than a specified point on the x-axis.

**Fig 6:**
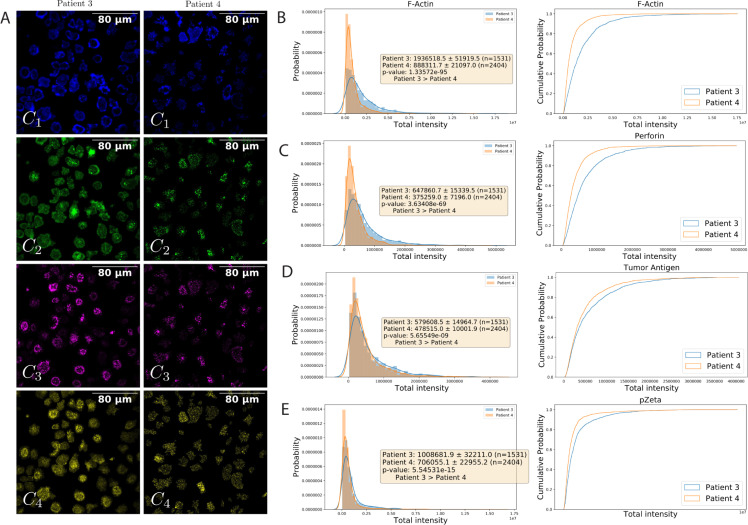
The total intensity in 4 channels. F-actin at row 1 (channel 1), perforin at row 2 (channel 2), tumor antigen at row 3 (channel 3), pZeta at row 4 (channel 4). (A) Shows one sample for each patient. The left side is for patient #3 and the right side for patient #4. In these images, the regions that do not belong to any predicted masks from ANNs are removed. Auto-contrast makes cells visible to human eyes (they do not affect real analysis). The (B), (C), (D), and (E) show the total intensity distribution and cumulative probability of two patients using fully trained networks across all channels for all counted cells from the evaluation phase. The figures also show the mean, variance, and the number of cells detected for each channel separately.

The total intensities in 4 channels are shown in Fig 6. F-actin at row 1 (channel 1), perforin at row 2 (channel 2), tumor antigen at row 3 (channel 3), pZeta at row 4 (channel 4). Fig 6A shows one sample for each patient. The left side is for patient #3 and the right side for patient #4. In these images, the regions that do not belong to any predicted masks from ANNs are removed. Fig 6B–6E shows the total intensity distribution and cumulative probability of two patients across all channels of the counted cells from the evaluation phase and using fully trained networks. The figures also show the mean, variance, and the number of cells detected for each channel separately.

In the perforin channel (Fig 6C), the total intensity of patient #3 is 647860.7 ± 15339.5, while patient #4’s is 375259.0 ± 7196.0. The t-test results show that the total intensity of patient #3 is significantly greater than the total intensity of patient #4. In the tumor antigen channel (Fig 6D), the total intensity of patient #3 is 579608.5 ± 14964.7, while patient #4’s is 478515.0 ± 10001.9. The t-test results show that the total intensity of patient #3 is significantly greater than the total intensity of patient #4. In the pZeta channel (Fig 6E), the total intensity of patient #3 is 1008681.9 ± 32211.0, while patient #4’s is 706055.1 ± 22955.2. The t-test results show that the total intensity of patient #3 is significantly greater than the total intensity of patient #4.

To determine the reproducibility of the results across different personnel and different densities of tumor antigen, we repeated the comparison of IS formation from patient #3 and patient #4 with different concentrations of tumor antigen (S1 File). Similarly, we found patient #3 had better IS quality, as determined by the 4 biomarkers, compared to patient #4 with both high and low densities of target tumor antigen.

Overall, our method implements the detection and segmentation of cells and quantifies four indicators related to CAR-T within the IS. We performed statistical analysis on the results and detected significant differences between the two patients in three different channels. We can assert that our method successfully quantifies CAR-T cells using IS data. This type of fast and reliable quantification is possible because of ML-based automation of the CAR IS image analysis.

## Discussion

This study developed an ML-based model for analyzing IS formed by the actual CAR-T cells from patients who participated in our clinical trials. The ML-based model effectively detects these CAR IS images in the presence of low contrast of cell boundaries, background noise (impurities), adhesion, and cell clustering. Specifically, we used ANNs to incorporate object detection and instance segmentation. The purpose of the proposed model is to extract the most useful information from the data to differentiate between different sets of data. The network output is flexible and produces bounding boxes, instance segmentation, contour outlines (borders), intensities of the borders, and intensities of the segmentations without borders.

Adoptive transfer of chimeric antigen receptor (CAR)-modified immune cells has shown remarkable success in clinical trials treating multiple refractory leukemia [39–42]. The cell therapy field invests considerable effort and funds into CAR optimization [43–46]. Several studies show a significant percentage of highly selected study patients do not respond to CAR-T therapy [4,31,47,48]. Furthermore, CAR therapy is associated with significant toxicity [49–53] and high cost [54]. The redundant efforts in CAR development in the cell therapy field could also be problematic in the long run. Thus, it is becoming imperative to predict which CAR construct will be most effective for a given cancer patient, and which patient will be a responder in a particular CAR treatment or ’off-the-shelf’ immune products (e.g., blinatumomab and anti-BCMA x anti-CD3 BITE agents). Recognizing the inadequacy of conventional tools, we investigate a new strategy for assessing CAR-T efficacy by quantification of CAR cell IS quality. In previous studies [4,8,9], we provide strong evidence that: 1) CAR-T cell IS quality (measured by CAR IS structure, function, and signaling) varies between CAR-T cells, 2) CAR co-stimulatory endodomains influence IS quality, 3) CAR-T IS quality correlates with antitumor activity both *in vitro* and *in vivo*, and 4) IS quality assay described in this study can distinguish between responder and non-responder.

In this study, we did not directly compare the SPE method with the conventional approaches. The conventional approaches to predict clinical outcomes in response to CAR therapy include multi-parametric flow cytometry, *in vitro* killing assays (e.g., short-term 4-h killing assay and long-term killing assay), cytokine productions by IsoPlexis [55,56], classic ELISA, and flow cytometry, RNA-Sequence of CAR-T cells [57], and other *in vitro* and *in vivo* animal models [4]. To ensure that transduced cells retain similar phenotypic and functional characteristics, researchers typically measure CAR-T cell growth kinetics and immunophenotype for 2–4 weeks after expansion [4,9]. After this, scientists examine whether transduction with CAR affects T-cell proliferation and cytokine production [18,23–25]. A standard 4-hour *Cr*^51^-release assay is the most common for evaluating short-term cytotoxicity [8]. A long-term killing assays evaluating long-term CAR-T cell activation, persistence, and proliferation in academia using a co-culture system [18,58,59]. A cell impedance system by xCELLigence Real-Time Cell Analysis (RTCA) is also common in industry [60]. *In vivo* strategies to assess homing, trafficking, persistence, and antitumor activity (e.g., severe combined immunodeficiency (SCID) mouse models and *in vivo* imaging systems [18–22]) are invaluable. Overall, currently available *in vitro* and *in vivo* strategies to evaluate CAR-T effectiveness in pre-clinical studies are time-consuming, expensive, labor-intensive, and inconsistent among laboratories. The low precision/reproducibility, low sensitivity, and low repeatability, and low accuracy represent a significant issue in the field of immunotherapy.

The study here investigated CAR-T IS quality as a potential proxy for CAR-T effectiveness biomarker, which is innovative and calls for further research on CAR IS. The specific contributions of this study include: 1) we showed that instant segmentation is most effective to automate the IS segmentation; 2) we used machine learning (ML) to quantify the CAR IS quality; 3) we provided a preliminary analysis to demonstrate the feasibility of predicting CAR-T efficacy using IS quality by an ML-based approach. Therefore, we propose that ML-based IS quality quantification can be used to potentially predict CAR efficacy to increase CAR treatment response. We applied ML-based methods to quantify the CAR cell IS, which initiated several validation processes to predict CAR efficacy in the future. Ultimately, we expect our findings to lay the groundwork for a low-cost, rapid, and high throughput ’Synapse Predicts Efficacy’ (SPE) testing system for basic and clinical research application.

However, the current study presents several limitations: 1) A small sample size was used in the current study. Specifically, we only evaluated two-patient samples from one DLBCL responder and one DLBCL non-responder from the Kappa-CAR-T cell treatment from our clinical trials. 2) Only tumor antigen on the glass-supported planar lipid bilayer was used. We did not include other co-stimulating and co-inhibitory molecules in the glass-supported planar lipid bilayer to mimic a real tumor cell directly isolated from patients. 3) This study has not included a tumor microenvironment (TME) factor. We did not include the TME in our current study due to the limited access to clinical resources. For example, obtaining the real tumor cells from these two patients who had undergone multiple biopsies is very challenging. 4) We did not evaluate the effects of intra-tumor heterogeneity on the IS quality. However, the automation of CAR IS quality analysis by an ML-based model developed in the current study represents a significant step in our persistent CAR IS study efforts.

In summary, this study provides a novel ML-based automated algorithm to quantify CAR-T IS formed by CAR-T cells from patients [4]. This study pioneers the measurement of CAR IS quality formed by patients’ CAR-T cells as a potential composite biomarker to predict antitumor activity in pre-clinical settings, which can potentially lead to the development of fast and easy tools to predict CAR-T cell effectiveness in cancer patients.

## Materials and methods

### CAR-T cells and stimulation of CAR-T cells on the glass-supported planar lipid bilayer

Human peripheral blood mononuclear cells (PBMCs) were purchased from New York Blood Center. The Rutgers University Institutional Review Board (IRB) approved the human blood related work in this study. Kappa-CAR-modified primary T (k.CAR-T) were derived from PBMCs isolated from patients who participated in the clinical trials (ClinicalTrials.gov NCT00881920 and ClinicalTrials.gov NCT01316146, which were conducted by Dr. Carlos Ramos from Baylor College of Medicine [BCM]). Formal consent was obtained for using k.CAR-T cells from patients who participated with clinical trials in Baylor College of Medicine. The Baylor College of Medicine Institutional Review Board (IRB) approved the k.CAR-T cells related work in this study. To stimulate the k.CAR-T cells and promote the CAR IS formation, we used the glass-supported planar lipid bilayer containing fluorescently labeled kappa protein, as described previously [8,32]. Specifically, planar lipid bilayers were prepared by fusing small liposome droplets with clean glass coverslips as described in [4]. Briefly, the liposome was trapped in a μ-Slide VI 0.4 chamber (Ibidi, Germany). Lipid bilayers were first blocked with 5% Casein for 30 minutes and then incubated with 6.3 nM Streptavidin (Life Technologies) for 20 minutes. Cells were activated on the lipids for 60 minutes before imaging. After being washed extensively with imaging buffer (HEPES-buffered saline), bilayers were incubated with biotinylated antibodies conjugated with Alexa Fluor dyes at room temperature for 30 minutes. After getting a second wash with imaging buffer, bilayers were blocked with 2.5 uM D-biotin to saturate the streptavidin-binding sites.

### Confocal imaging on the planar lipid bilayer

k.CAR-T cells were stimulated on lipid bilayers containing fluorescently labeled Kappa proteins. Cells were stained by fluorescently conjugated antibodies against perforin (deltaG9, Thermo), pZeta (phosphor-Y83, Abcam), as described previously [8]. F-actin was stained by Alexa Fluor 532-conjugated or Alexa Fluor 405-conjugated phalloidin (Life Technologies, CA, USA). A Nikon advanced confocal microscope system A1R HD25 (Nikon, Japan) was used to obtain confocal image data.

### Patient characteristics

Patient #3 was a 53-year-old male with a history of follicular lymphoma transformed to diffuse large B cell lymphoma, treated initially with R-CHOP × 6, with PR; then R-IE × 3, followed by ASCT, with CR, and Rituximab maintenance for two years. Patient #3 later relapsed (in a single node in the neck) and had that lymph node resected, and later had a second relapse (in pelvic lymph nodes), at which point he was enrolled on our CHARKALL protocol in the previous publication [31]. Patient #3, defined as a responder in this study, had a transient CR to the k.CAR-T cells and for many years now have had stable disease.

Patient #4 was a 69-year-old male with DLBCL, who was initially treated with R-CHOP × 6 followed by ASCT, with CR, but had an early relapse (as diffuse large B cell lymphoma); treated with a couple of salvage therapies (including bendamustine and ibrutinib), with progressive disease, at which point he was enrolled on our CHARKALL protocol. Patient #4, whose unique number is #15 in the previous publication [31], had no response to the k.CAR-T cells and therefore is defined as a non-responder in this study. The exact characteristics of both patients are presented in Table 2.

### Total fluorescence intensity (TFI) quantification

**Fig 7:**
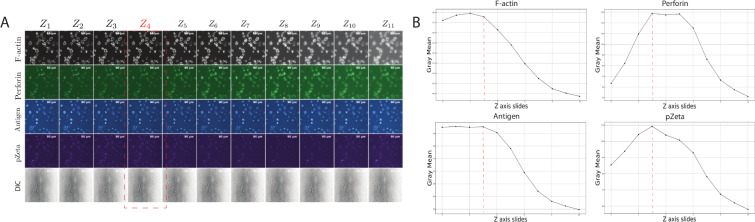
Successful image data extraction in a python environment. (A) Is a sample of 11 Z slides with five channels: F-actin at row 1 (channel 1), perforin at row 2 (channel 2), tumor antigen at row 3 (channel 3), pZeta at row 4 (channel 4) and, the DIC of the cells at row 5 (channel 5). To have clear representations of the cells in the figure, colormap filters are added to the original grayscale images: F-actin received ’RdGy_r’ colormap, perforin received ’PRGn_r’ colormap, tumor antigen received ’RdBu_r’ colormap, pZeta received ’PuOr_r’ colormap and, the DIC received ’binary’ colormap. The colormaps [61] are only used for representation purposes and do not affect the evaluation of the IS. (B) Plots the mean intensity values for grayscale images through Z slides for all channels.

Our IS image dataset contains 156 three-dimensional (3D) images in total. Each image contains 5 channels: F-actin (channel 1), perforin (channel 2), tumor antigen (channel 3), pZeta (channel 4), and the differential interference contrast (DIC) of the cells (channel 5). Each channel has a different number of slices (3D image stack for each channel, typically not more than 15 slices). First, we identified slice Z as the location to find the maximum intensity within the tumor antigen channel. This is done because the focal plane of the CAR-T IS is best captured where the tumor antigen clusters on the glass-supported planar lipid bilayer (Fig 7).

We extract slice Z information from the F-actin channel as the reference image to find each cell contour. We then apply the contours generated by the F-actin channel into other channels, including perforin (channel 2), tumor antigen (channel 3), pZeta (channel 4), and the DIC of the cells (channel 5). The ROIs for each individual cell are calculated by the areas of the contours generated by the F-actin channel. In this study, we have implemented an effective method of detecting cells, segmenting them, and getting their masks. After obtaining single-cell contours according to their masks, we apply them with the grayscale images derived from the original image to get a total fluorescence intensity (TFI).

### Antibodies and reagents

Alex Fluor 647 (AF647) Goat anti-human IgG F(ab’)2 fragment antibody was purchased from Jackson ImmunoResearch (West Grove, PA, USA). Purified anti-CD247 (also known as T-cell surface Glycoprotein CD3 Zeta Chain, CD3) antibody (clone 6B10.2, BioLegend), PE- or APC-conjugated anti-human CD3 antibody (clone OKT3, BioLegend), FITC or BV 510-conjugated anti-human CD56 antibody (clone HCD56, BioLegend) were purchased from BioLegend (San Diego, CA, USA).

### CAR-T cell segmentation on the glass-supported planar lipid bilayer

To obtain the quantified parameters of CAR-T IS, we used nuclei segmentation with multi-scale cell instance segmentation. Nuclei segmentation is the process of the detection and extraction [27] of CAR-T cells from planar lipid bilayer images. The image processing was conducted using Python, and OpenCV 2.0 libraries [62]. Multi-scale cell instance segmentation uses deep neural network frameworks from the PyTorch library [63] on a standard workstation with NVIDIA GTX1060 GPU. The CAR-T detection encompasses two modules. The first module detects the different bounding boxes (BBox Generation) and, the second module focuses on individual cell segmentation (instance segmentation).

**Fig 8:**
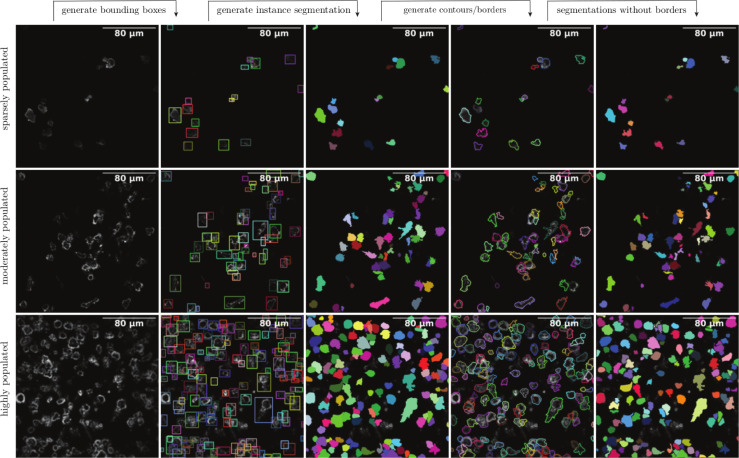
The outputs of multi-scale cell instance segmentation. For illustration, we use three different images in three different rows. The first row is for a sparsely populated image, the second row is for a moderately populated image, and the third row is for a highly populated image. The framework contains two modules: (a) bounding box detection module and (b) individual cell segmentation module. The bounding box detection outputs the bounding boxes over each detected cell. The bounding box determines an object by indicating the top-left, top-right, bottom-left, bottom-right, and center points, respectively. The bounding boxes are used to create patches of cells, used for instance segmentation. The instance segmentation masks can be used to create borders (contours) and segmentations without borders, which are inside the areas of the segmented objects.

Fig 8 shows the outputs of multi-scale cell instance segmentation. In this image, we use three different types of images in three different rows. The first row is for a sparsely populated image, the second is for a moderately populated image, and the third row is for a highly populated image. The instance segmentation masks can create borders (contours) and segmentations without borders inside the segmented objects. Based on the intrinsic nature of microscopical images, each of these outputs or their combinations can be used for statistical analysis. For CAR-T quantification within the IS, our experience shows that instance segmentation is the best criterion.

### Bounding box generation

We use multi-scale cell instance segmentation to find the bounding boxes over cells [28,29]. In this framework, keypoint detection [64] is utilized to determine the top-left, top-right, bottom-left, bottom-right, and center for each cell separately. Keypoint detection, operates on four scale detections *s_i_*, *i* = 1,2,3,4, and several steps to output desired rectangles. First, disks (circular frames) are placed on the image based on different scales. From the disks, five heatmaps are placed inside the disk to predict the possibility of keypoint locations. For the heatmaps, offset maps are used to extract the local maxima for each heatmap disc. For each offset map, two channels are used for each keypoint to show the displacements of keypoints both in the horizontal and vertical directions. The Hough accumulators use the heatmaps and offsetmap for Hough voting [64–66], which aggregates the keypoint groups at scales *s_i_*, *i* = 1,2,3,4. Any pair of diagonal points and any three points can create bounding boxes from the possible keypoint groups. In the end, the non-maximum suppression (NMS) operation [67] is applied to prevent the detection of the same object multiple times.

### Cell segmentation

There are two types of image segmentation: semantic and instance segmentation. The semantic segmentation methods, such as TIAM-HT [68,69], are designed to treat multiple objects within a single category as one entity. On the other hand, instance segmentation methods treat individual objects within a single category as different entities. The methodology used in this paper performs instance segmentation on the cells. Not only does it find the overall masks of the cells (semantic segmentation), but it also distinguishes each cell separately (instance segmentation). Individual cell segmentation is performed on patches of cells obtained from bounding boxes related to individual cells for input images. The feature maps from low levels and feature maps from high levels are combined to take advantage of semantic information for high and low-level details. This methodology is motivated by U-Net [35] and is useful for nuclei segmentation. Cell patches are created from shallow layers of deep neural networks, and then a bottom-up segmentation is performed on the patches. Note that the module for cell segmentation uses different feature maps than the network used in the bounding box generation module. This design helps to prevent interference of neighboring cells. Specifically, focusing on unique patches of cells helps with segmenting irregular shapes.

### Accuracy analysis

There are two kinds of accuracy analysis: one is detecting CAR-T cells with bounding boxes [70–72], and the other is instance segmentation of the detected cells [73–75]. The ground truth bounding boxes from training sets are used to train the segmentation module. To test the method, we first generate bounding boxes with keypoints detection, which is then used for instance segmentation. For the evaluation metric of instance segmentation, average precision (AP) at box-level and intersection over union (IOU) with thresholds of 0.5 and 0.7 are deployed. Average precision (AP) of 0.5 and 0.7 are deployed for bounding boxes. These metrics are standard methods to evaluate bounding box generation, and instance segmentation [76,77].

### Statistical analysis

Statistical significance was determined by using two-tailed independent t-test samples. For this purpose, two separate sets of independent and identically distributed samples are obtained, one from each of the two populations being compared. This is a two-sided test for the null hypothesis that two independent samples have identical average values (expected). Usually, we refer to statistically significant as P < 0.05 and statistically highly significant as P < 0.001 (less than one in a thousand chance of being wrong). In this study, the t-test is used to determine whether the data distributions of two patients are significantly different.

## Supporting information

S1 Data Excel spreadsheet containing, in separate sheets, the underlying numerical data for Figs 1B, 7C, 7D, 8B, 8C, 8D and 8E. (XLSX)

S1 File In Vitro Machine Learning-Based CAR-T Immunological Synapse Quality Measurements Correlate with Patient Clinical Outcomes. (DOCX)
