# Supplementary material for: In vitro machine learning-based CAR T immunological synapse quality measurements correlate with patient clinical outcomes
Source: PLoS Comput Biol. 2022 Mar 18;18(3):e1009883. doi: 10.1371/journal.pcbi.1009883 (PMC8955962; doi:10.1371/journal.pcbi.1009883)
Supplement: S1 File — (DOCX) [file pcbi.1009883.s002.docx]

**S1 File. In Vitro Machine Learning-Based CAR-T Immunological Synapse Quality Measurements Correlate with Patient Clinical Outcomes**

**Supplementary Data**

To determine whether the results are reproducible across different personnel with different tumor antigen concentrations, we repeated the comparison of immunological synapse quality from patient #3 (a long-term responder for kappa-CAR T therapy) and patient #4 (a non-responder for kappa-CAR-T therapy). The supporting information Fig A-D results show that at low densities of kappa protein using 10ng/well, we could replicate the immunological synapse quality trend between patient #3 and patient #4. We found that pZeta, Perforin, and F-actin were all significantly increased compared to patient #4, consistent with our prior results.


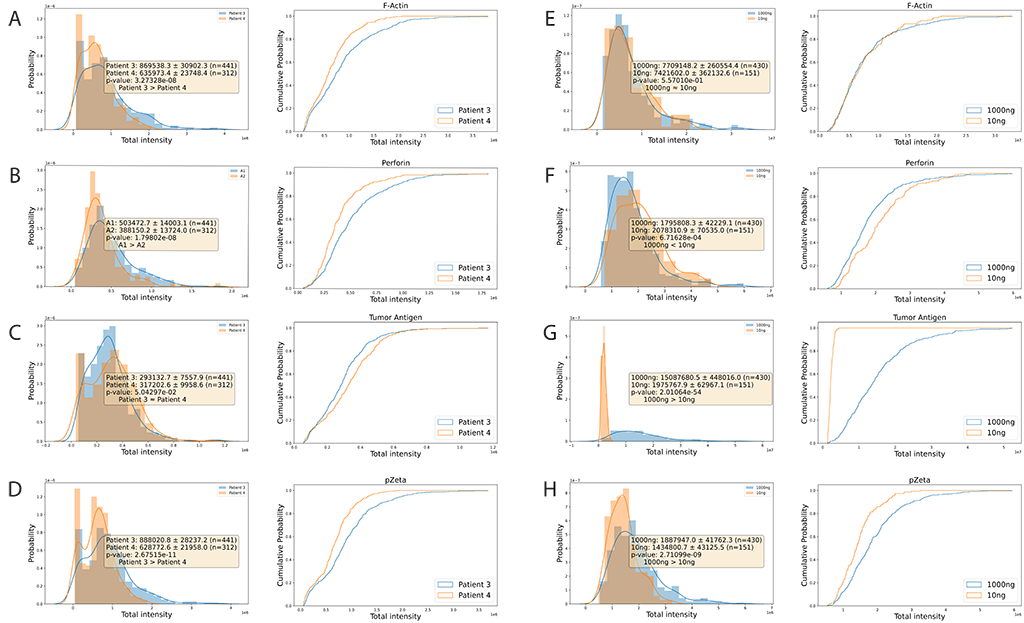


**Fig A-H.** A repeat of the immunological synapse quality comparison between patient #3 vs. patient #4. The immunological synapse quality results were reproducible using a low density of tumor antigen closer to the physiological conditions. The chart shows the total intensity of 4 channels. F-actin at row 1 (channel 1), Perforin at row 2 (channel 2), Tumor antigen-Kappa protein at row 3 (channel 3), and pZeta at row 4 (channel 4). (A-D) show the comparison between patient #3 (blue) and patient #4 (orange) samples. (E-H) show the comparison between high-density 1000ng/well (blue) and low-density 10ng/well (orange). The figure legends show the mean, variance, and the number of cells detected for each channel separately.

It has been shown that lower densities of tumor antigen weaken CAR-T cell activation, function, and persistence, which mediates CAR-T cell resistance [1]. We expect lower tumor antigen density leads to lower immunological synapse formation between CAR-T cells and target antigen. To demonstrate this, we performed a lipid bilayer assay using patients' Kappa-CAR-T cells with two different densities of Kappa tumor antigen on the glass-supported planar lipid bilayer system (Fig E-H). We show that higher tumor antigen densities (1000 ng/well) correspond with an increased number of cells forming immunological synapses and higher quality of immunological synapse (F-actin, pZeta, and tumor antigen), compared to lower tumor antigen densities (10 ng/well) (Fig E-H). Together, these show that our method readily detects immunological synapse quality differences at high and low densities. Moreover, we detected superior immunological synapse quality in patient #3 compared to patient #4 at physiological-relevant densities, suggesting a potential approach to utilize immunological synapse quality as a prognostic indicator of clinical response.

**References**

1. Spiegel JY, Patel S, Muffly L, Hossain NM, Oak J, Baird JH, et al. CAR T cells with dual targeting of CD19 and CD22 in adult patients with recurrent or refractory B cell malignancies: a phase 1 trial. Nature medicine. 2021;27(8):1419-31.
